# Simultaneous Pathoproteomic Evaluation of the Dystrophin-Glycoprotein Complex and Secondary Changes in the *mdx-4cv* Mouse Model of Duchenne Muscular Dystrophy

**DOI:** 10.3390/biology4020397

**Published:** 2015-06-10

**Authors:** Sandra Murphy, Michael Henry, Paula Meleady, Margit Zweyer, Rustam R. Mundegar, Dieter Swandulla, Kay Ohlendieck

**Affiliations:** 1 Department of Biology, Maynooth University, Maynooth, Co. Kildare, Ireland; E-Mail: sandra.murphy@nuim.ie; 2 National Institute for Cellular Biotechnology, Dublin City University, Dublin 9, Ireland; E-Mails: michael.henry@dcu.ie (M.H.); paula.meleady@dcu.ie (P.M.); 3 Department of Physiology II, University of Bonn, Bonn D-53115, Germany; E-Mails: margit.zweyer@ukb.uni-bonn.de (M.Z.); mundegar@yahoo.com (R.R.M.); dieter.swandulla@ukb.uni-bonn.de (D.S.)

**Keywords:** dystroglycan, dystrophin, dystrophinopathy, Na^+^/K^+^-ATPase, myozenin, organelle proteomics, periostin, sarcoglycan, syntrophin, tubulin

## Abstract

In skeletal muscle, the dystrophin-glycoprotein complex forms a membrane-associated assembly of relatively low abundance, making its detailed proteomic characterization in normal *versus* dystrophic tissues technically challenging. To overcome this analytical problem, we have enriched the muscle membrane fraction by a minimal differential centrifugation step followed by the comprehensive label-free mass spectrometric analysis of microsomal membrane preparations. This organelle proteomic approach successfully identified dystrophin and its binding partners in normal *versus* dystrophic hind limb muscles. The introduction of a simple pre-fractionation step enabled the simultaneous proteomic comparison of the reduction in the dystrophin-glycoprotein complex and secondary changes in the *mdx-4cv* mouse model of dystrophinopathy in a single analytical run. The proteomic screening of the microsomal fraction from dystrophic hind limb muscle identified the full-length dystrophin isoform Dp427 as the most drastically reduced protein in dystrophinopathy, demonstrating the remarkable analytical power of comparative muscle proteomics. Secondary pathoproteomic expression patterns were established for 281 proteins, including dystrophin-associated proteins and components involved in metabolism, signalling, contraction, ion-regulation, protein folding, the extracellular matrix and the cytoskeleton. Key findings were verified by immunoblotting. Increased levels of the sarcolemmal Na^+^/K^+^-ATPase in dystrophic leg muscles were also confirmed by immunofluorescence microscopy. Thus, the reduction of sample complexity in organelle-focused proteomics can be advantageous for the profiling of supramolecular protein complexes in highly intricate systems, such as skeletal muscle tissue.

## 1. Introduction

Although the total number of proteins in contractile tissues is not precisely known [1], the findings from systematic cataloguing studies suggest that several thousand protein species constitute the protein repertoire of the skeletal muscle proteome [2,3,4,5,6]. Importantly, individual skeletal muscles contain a heterogeneous mixture of slow- and fast-twitching fibre types [7] with considerably different protein constellations [8,9,10]. The highly complex and species-selective combination of type I, type IIa, type IIb and type IId/x fibres, as well as a variety of hybrid fibres and satellite cell populations, changes substantially during development, muscle transformation, physiological adaptations, pathological alterations, and the natural aging process [11]. The biochemical diversity of muscles is reflected by an extensive molecular heterogeneity on the level of protein isoform expression patterns. Proteomic surveys indicate that several hundred proteins are differentially expressed in contractile tissue with a differing fibre specification [12,13,14,15]. This includes, especially, the contractile and regulatory elements of the actomyosin apparatus, such as the many isoforms of myosin heavy chains, myosin light chains, actins, troponins and tropomyosins, but also many metabolic enzymes, signalling proteins, and ion-handling proteins [16,17,18].

This molecular heterogeneity and the wide dynamic concentration range of different muscle-associated protein families represent a challenging technical obstacle to the elucidation of the molecular complexity of the skeletal muscle proteome [19]. One potential option to overcome some aspects of these analytical difficulties is the reduction of protein sample complexity through the introduction of pre-fractionation steps in the proteomic workflow [20,21,22]. Organelle proteomic approaches have been successfully applied to studying skeletal muscle tissues [23], including the nucleus [24], the cytosol [25,26], the sarcolemma [27], the sarcoplasmic reticulum [28,29] and mitochondria [30,31,32]. Building on the previous gel-based analysis of the contractile apparatus-depleted fraction from dystrophic muscle [33], we carried out here the comprehensive label-free LC-MS/MS analysis of the microsomal fraction from dystrophin-deficient hind limb muscle. The main underlying objective of this proteomic study was to attempt the simultaneous identification of the dystrophin-glycoprotein complex and the systematic cataloguing of downstream changes in low copy number proteins and their potential involvement in X-linked muscular dystrophy.

Duchenne muscular dystrophy is a monogenic disorder that is characterized by primary abnormalities in the dystrophin gene that cause the almost complete absence of the Dp427 isoform of this membrane cytoskeletal protein in muscle tissues [34]. Animal models of X-linked muscular dystrophy have been instrumental in studying fundamental aspects of the molecular pathogenesis of dystrophinopathies [35,36,37] and the evaluation of experimental treatment strategies [38]. The *mdx-4cv* mouse is lacking dystrophin due a mutation in exon 53 and exhibits considerably less revertant fibres as compared to the conventional *mdx* mouse that has a mutation in exon 23 [39,40]. Previous proteomic studies of dystrophic muscles using crude tissue extracts have established a variety of secondary changes in muscular dystrophy, such as alterations in proteins involved in excitation-contraction coupling, ion homeostasis, the contraction-relaxation cycle, signalling, metabolism and the cellular stress response [41,42,43,44,45]. Building on these extensive proteome-wide data sets on global alterations in dystrophin-deficient muscle tissues [46], our new approach has employed the microsomal membrane fraction and identified simultaneously the drastic reduction in the dystrophin-glycoprotein complex and a large number of secondarily affected proteins in X-linked muscular dystrophy in a single subproteomic analytical run.

## 2. Experimental Section

### 2.1. Chemicals and Materials

Analytical grade reagents and materials for the mass spectrometry-based proteomic profiling of *mdx-4cv* *versus* wild type hind limb muscle were obtained from GE Healthcare (Little Chalfont, UK) and Bio-Rad Laboratories (Hemel-Hempstead, UK). Ultrapure acrylamide stock solutions were attained from National Diagnostics (Atlanta, GA, USA). Sequencing grade modified trypsin and Lys-C were from Promega (Madison, WI, USA). Whatman nitrocellulose transfer membranes were purchased from Invitrogen (Carlsbad, CA, USA). The chemiluminescence substrate and protease inhibitors were obtained from Roche Diagnostics (Mannheim, Germany). Primary antibodies were purchased from Abcam, Cambridge, UK (ab2818 to the fast SERCA1 isoform of the sarcoplasmic reticulum Ca^2+^-ATPase; ab92721 to myosin light chain isoform MLC2; ab21754 to the β-subunit of tubulin; ab58475 to the α-subunit of the Na^+^/K^+^-ATPase; ab88184 to myozenin 1; ab52488 to lactate dehydrogenase, and ab110330 to pyruvate dehydrogenase), Santa Cruz Biotechnology, Dallas, TX, USA (sc-25607 to myoglobin, and sc-32322 to vimentin), NovoCastra, Leica Biosystems, Newcastle Upon Tyne, UK (NCL-Dys2 to the carboxy terminus of dystrophin isoform Dp427) and Novus Biologicals, Cambridge, UK (NBP1-30042 to the matricellular protein periostin). Chemicon International (Temecula, CA, USA) provided peroxidase-conjugated secondary antibodies. For immunofluorescence microscopy, normal goat serum, goat anti-rabbit Alexa Fluor 488, and goat anti-mouse IgG RRX (Rhodamine Red-X) were purchased from Molecular Probes, Life Technologies (Darmstadt, Germany) and Jackson ImmunoResearch (West Grove, PA, USA), respectively. The embedding medium Fluoromount G was from Southern Biotech (Birmingham, AL, USA). A variety of other general chemicals, including bis-benzimide Hoechst-33342, were obtained from Sigma Chemical Company (Dorset, UK).

### 2.2. Animal Model of X-Linked Muscular Dystrophy

The conventionally used *mdx* mouse model represents a naturally occurring mutant, in which the primary genetic mutation is a base substitution in exon 23 of the dystrophin gene. This substitution introduces a premature stop codon, and so in analogy to Duchenne patients, the *mdx* mouse model almost completely lacks the full-length dystrophin isoform Dp427 [37]. However, this study utilised an alternative model of dystrophinopathy, the *mdx-4cv* mouse [40], which has been generated using N-ethylnitrosourea [47]. Chemical mutagenesis was shown to result in a C to T transition at base 7916 in exon 53, generating an ochre codon [48]. The *mdx-4cv* model has approximately 10-fold fewer revertant fibres than the *mdx* model and represents a more precise genocopy of the human pathology [39]. The fact that very few dystrophin-positive revertant fibres exist in *mdx-4cv* skeletal muscles [49] makes this animal model attractive for the assessment of novel therapeutic approaches. Previous studies have evaluated the efficiency of antisense oligomer-induced exon skipping following intramuscular injections into the *mdx-4cv* *tibialis anterior* muscle [50] and determined the stability of dystrophin expression after lentiviral vector injection into neonatal *mdx-4cv* muscles [51]. The analysis of proteome-wide changes in this animal model should, therefore, provide a comprehensive list of altered proteins that may be useful as therapy-monitoring biomarkers in the future evaluation of new experimental therapies. In order to study differential protein expression patterns due to deficiency in dystrophin, hind leg muscles from 6-month old dystrophic *mdx-4cv versus* age-matched control C57BL6 mice were studied. Fresh tissue samples were acquired from the Bioresource Unit of the University of Bonn [52]. Cardiac tissues were used for control purposes. The mice were kept under standard conditions according to German and Irish legislation on the use of animals in experimental research. The animals were sacrificed by cervical dislocation and muscle tissues were immediately isolated. The specimens used for proteomic analysis were quick-frozen in liquid nitrogen and stored at −80 °C prior to analysis.

### 2.3. Subcellular Fractionation of Muscle Homogenates

Since this organelle proteomic study employed a differential centrifugation approach to reduce sample complexity prior to label-free mass spectrometry, a relatively large volume of starting material was a pre-requisite as compared to crude whole tissue proteomics. For this reason, proteomic analysis was conducted on combined muscles from the entire mouse hind limb. The subcellular fractionation of skeletal muscle homogenates was based on previously optimised protocols for the separation of distinct membrane-enriched parts of this highly complex and heterogeneous type of contractile tissue [33]. Muscle specimens (~750 mg wet weight) from the hind leg muscles of 6-month old *mdx-4cv* mice (*n* = 4) and age-matched normal mice (*n* = 4) were finely chopped and homogenised in 10 volumes of homogenisation buffer (20 mM sodium pyrophosphate, 20 mM sodium phosphate, 1 mM MgCl_2_, 0.303 M sucrose, 0.5 mM EDTA, pH 7.0), using a hand-held IKA T10 Basic Homogeniser (IKA Labortechnik, Staufen, Germany). To prevent degradation of sensitive skeletal muscle proteins, the buffer was supplemented with a protease inhibitor cocktail from Roche Diagnostics [41]. Crude protein extracts were gently shaken at 8 °C for 2 h using a Thermomixer from Eppendorf (Hamburg, Germany). The muscle homogenates were centrifuged at 14,000 g for 15 min at 4 °C. For comparative immunoblotting studies, crude cardiac extracts were isolated by the same procedure using 5-month old normal *versus* age-matched *mdx-4cv* hearts. For the isolation of microsomes, the supernatant was pelleted at 100,000 g for 1 h at 4 °C using an Optima L-100 XP ultracentrifuge from Beckman Coulter, Inc. (Fullerton, CA, USA) [33]. Microsomal pellets were resuspended in an appropriate volume of homogenisation buffer and characterized by label-free LC-MS/MS analysis.

### 2.4. Sample Preparation for Label-Free Liquid Chromatography Mass Spectrometric Analysis

Microsomal fractions were pre-treated with the Ready Prep 2D clean up kit from Bio-Rad Laboratories. The pellets obtained from this procedure were re-suspended in label-free solubilisation buffer (6 M urea, 2 M thiourea, 10 mM Tris, pH 8.0 in LC-MS grade water). Full re-suspension was aided by vortexing and sonication of the protein suspensions [52]. Protein concentrations were determined by the Bradford assay protocol [53]. Protein suspension volumes were initially equalised with label-free solubilisation buffer. Samples were reduced with 10 mM DTT for 30 min at 37 °C and were alkylated with 25 mM iodoacetamide in 50 mM ammonium bicarbonate for 20 min in the dark at room temperature [52]. To quench any unreacted iodoacetamide and thus limit potential alkylation of trypsin, samples were reduced with a further 10 mM DTT for 15 min in the dark at room temperature. Controlled proteolytic digestion was conducted using a combination of the enzymes Lys-C and trypsin. The initial digestion was carried out with sequencing-grade Lys-C at a ratio of 1:100 (protease:protein) for 4 h at 37 °C, followed by dilution with four times the initial sample volume in 50 mM ammonium bicarbonate. Samples were subsequently digested with sequencing-grade trypsin at a ratio of 1:25 (protease:protein) overnight at 37 °C. Digestion was halted by acidification with 2% trifluoroacetic acid (TFA) in 20% acetonitrile (ACN) (3:1 (v/v) dilution). Peptide suspensions were purified using Pierce C18 Spin Columns from Thermo Fisher Scientific (Dublin, Ireland) and the resulting peptide samples were dried through vacuum centrifugation and suspended in loading buffer consisting of 2% ACN and 0.05% TFA in LC-MS grade water. Samples were vortexed and sonicated to ensure an even suspension of peptides.

### 2.5. Label-Free Liquid Chromatography Mass Spectrometric Analysis

An Ultimate 3000 NanoLC system (Dionex Corporation, Sunnyvale, CA, USA) coupled to a Q-Exactive mass spectrometer (Thermo Fisher Scientific) was used for the label-free liquid chromatography mass spectrometric (LC-MS/MS) analysis of *mdx-4cv versus* wild type samples. Peptide mixtures (3 µL) were loaded by an autosampler onto a C18 trap column (C18 PepMap, 300 µm id *×* 5 mm, 5 µm particle size, 100 A pore size; Thermo Fisher Scientific). The trap column was switched on-line with an analytical Biobasic C18 Picofrit column (C18 PepMap, 75 µm id × 50 cm, 2 µm particle size, 100 A pore size; Dionex). Peptides generated from muscle proteins were eluted using the following gradient (solvent B: 80% (v/v) ACN and 0.1% (v/v) formic acid in LC-MS grade water): 0% solvent B for 10.5 min, 2% solvent B for 110 min, 40% solvent B for 2.5 min, 90% solvent B for 9 min and 2% solvent B for 43 min. The column flow rate was set to 0.25 µL/min. Data were acquired with Xcalibur software (Thermo Fisher Scientific). The mass spectrometer was operated in positive mode and data-dependent mode and was externally calibrated. Survey MS scans were conducted in the Q-Exactive mass spectrometer in the 300–1700 *m/z* range with a resolution of 140,000 (*m/z 200*) and lock mass set to 445.12003. CID (collision-induced dissociation) fragmentation was carried out with the fifteen most intense ions per scan and at 17,500 resolution. Within 30 s, a dynamic exclusion window was applied. An isolation window of 2 *m/z* and one microscan were used to collect suitable tandem mass spectra.

### 2.6. Quantitative Proteomic Profiling by Label-Free LC-MS/MS Analysis

Processing of the raw data generated from LC-MS/MS analysis was carried out using Progenesis QI for Proteomics software (version 3.1; Non-Linear Dynamics, a Waters company, Newcastle upon Tyne, UK). Data alignment was based on the LC retention time of each sample. Any drift in retention time was allowed for, giving an adjusted retention time for all runs in the analysis [54]. The sample run that yielded the most features (peptide ions) was selected as a reference run. The retention times of all the other runs were aligned to this reference run and peak intensities were normalised. The data was filtered using certain criteria prior to exporting the MS/MS data files to Proteome Discoverer 1.4 (Thermo Scientific); (i) peptide features with ANOVA ≤ 0.05 between experimental groups, (ii) mass peaks with charge states from +1 to +5 and (iii) greater than one isotope per peptide [55]. A PepXML generic file was generated from all exported MS/MS spectra from Progenesis software. This file was used for peptide identification using Proteome Discoverer 1.4 against Mascot (version 2.3, Matrix Science, Boston, MA, USA) and Sequest HT (SEQUEST HT algorithm, licence Thermo Scientific, registered trademark University of Washington, USA) and searched against the UniProtKB-SwissProt database (taxonomy: *Mus musculus*). The following search parameters were used for protein identification: (i) peptide mass tolerance set to 10 ppm, (ii) MS/MS mass tolerance set to 0.02 Da, (iii) up to two missed cleavages were allowed, (iv) carbamidomethylation set as a fixed modification and (v) methionine oxidation set as a variable modification [56]. For re-importation back into Progenesis LC-MS software for further analysis, only peptides with either ion scores of 40.00 or more (from Mascot) and peptides with XCorr scores >1.9 for singly charged ions, >2.2 for doubly charged ions and >3.75 for triply charged ions or more (from Sequest HT) were selected. Importantly, the following criteria were applied to assign a muscle-associated protein as differently expressed: (i) an ANOVA score between experimental groups of *≤*0.05, and (ii) proteins with *≥*2 peptides matched. Standard bioinformatics software programmes were used to group proteins based on their molecular function and to determine potential interaction patterns between altered proteins. These analyses were performed on the total protein content of wild type microsomal extracts, and on the MS-identified proteins with a changed abundance in the microsomal *mdx-4cv* fractions. This bioinformatics analysis was performed with the PANTHER database of protein families for the cataloguing of molecular functions [57,58] and the STRING database of known and predicted protein interactions that include direct physical and indirect functional protein associations [59,60].

### 2.7. Verification of Key Proteomic Findings by Comparative Immunoblot Analysis

To illustrate successful isolation of the microsomal fraction from crude muscle homogenates and to authenticate the key findings from the proteomic profiling of the accessible protein constellation of normal *versus* dystrophic muscle, immunoblotting with antibodies against select muscle proteins was employed. Routine 1D gel electrophoresis and immunoblot analysis was carried out using well-established methods with crude extracts from skeletal and cardiac muscles, as well as microsomal preparations from skeletal muscle [28]. Electrophoretic separation of proteins was performed with standard 10% polyacrylamide gels, followed by wet transfer at 100 V for 70 min at 4 °C to Whatman Protan nitrocellulose sheets in a Transblot Cell from Bio-Rad Laboratories. To prevent non-specific antibody binding, membranes were blocked for 1 h with a milk protein solution (5% (w/v) fat-free milk powder in 10% phosphate-buffered saline). Membranes were incubated with diluted primary antibodies overnight at 4 °C with gentle agitation. The membranes were then washed with the milk protein solution twice for 10 min, followed by incubation for 1 h with peroxidise-conjugated secondary antibodies. The antibody concentrations were optimised to facilitate immunochemical protein detection while preventing non-specific interactions. This was followed by several washing steps with the milk protein solution and with 10% phosphate-buffered saline. Visualisation of antibody-labelled protein bands was achieved using the enhanced chemiluminescence method in accordance with the manufacturer’s guidelines. Densitometric scanning and statistical analysis of immunoblots was executed using a HP PSC-2355 scanner and ImageJ software (NIH, Bethesda, MD, USA) along with Graph-Pad Prism software (San Diego, CA, USA), in which a *p* value < 0.05 was deemed to be statistically significant.

### 2.8. Immunofluorescence Microscopy

For the immunofluorescence microscopical comparison of normal *versus* dystrophic *gastrocnemius* muscles, freshly dissected tissue specimens were quick-frozen in liquid nitrogen-cooled isopentane and 10 μm sections cut in a cryostat [41]. For routine immuno-labelling, tissue sections were fixed in a 1:1 (v/v) mixture of methanol and acetone for 10 min at room temperature. For dystrophin immuno-staining, unfixed cryosections were boiled in phosphate-buffered saline for 5 min as previously described in detail [61]. Tissue sections were permeabilised in 0.1% (v/v) Triton X-100 for 10 min and then blocked with 1:20 diluted normal goat serum for 30 min at room temperature. Primary antibodies to dystrophin and the Na^+^/K^+^-ATPase were diluted 1:20 and 1:10, respectively, in phosphate-buffered saline for overnight incubation at 4 °C. Specimens were carefully washed and then incubated with fluorescently-labelled secondary antibodies, using either 1:200 diluted anti-rabbit Alexa Fluor 488 antibody or 1:200 diluted anti-mouse RRX antibody for 45 min at room temperature. Potential auto-fluorescence was quenched by incubation in 1% (w/v) Sudan Black for 10 min. Nuclei were counter-stained with 1 μg/mL bis-benzimide Hoechst 33342. Antibody-labelled tissue sections were embedded in Fluoromount G medium and viewed under a Zeiss Axioskop 2 epifluorescence microscope equipped with a digital Zeiss AxioCam HRc camera (Carl Zeiss Jena GmbH, Jena, Germany).

## 3. Results and Discussion

The majority of previous cataloguing or differential profiling studies of skeletal muscle tissues have failed to detect or only partially identified the main members of the dystrophin-glycoprotein complex [46]. This could be at least partially due to (i) the large size of this supramolecular assembly, (ii) its tight association with the muscle surface membrane and (iii) its relatively low abundance as compared to the entire muscle proteome. In fibre extracts, approximately half of the total protein fraction is represented by the actomyosin complex in conjunction with its regulatory proteins including troponins and tropomyosins and a variety of auxiliary filament proteins, such as nebulins, titins, the Z-disc complex and the M-zone assembly [18]. In biochemical studies of the total membrane fraction, the majority of vesicle-associated proteins derive from the abundant cohort of mitochondria and the extensive sarcoplasmic reticulum system with longitudinal tubules and terminal cisternae. On the level of the muscle cytosol, the most plentiful class of proteins are the enzymes that provide the glycolytic pathway [25]. It is, therefore, not surprising that shotgun proteomics of various skeletal muscles has not succeeded in the systematic cataloguing of all members of the dystrophin complex [2,3,4,5] and that comparative studies using total extracts from dystrophic fibres have been unsuccessful in the comprehensive identification of dystrophin and its associated glycoproteins [41,42,43,44,45,62,63]. Alternative strategies have employed extensive purification steps, such as a combination of ion exchange chromatography, lectin agglutination and density gradient centrifugation [27] or immuno precipitation protocols [64,65,66]. The proteomic quantitation of select peptides representing dystrophin within a complex protein mixture has been carried out by combining stable isotope labelled dystrophin as a spike-in standard, one-dimensional gel electrophoretic separation, and mass spectrometry [67]. However, these elaborate purification steps may introduce a large number of potential analytical artefacts prior to mass spectrometric analysis and thus do not allow the proper comparative analysis of secondary alterations downstream from the primary abnormality in muscular dystrophy [19].

To overcome these technical obstacles, we have introduced here a simple pre-fractionation step to enrich the dystrophin complex in the microsomal fraction from normal *versus* dystrophic muscle preparations. This enabled the simultaneous proteomic evaluation of the reduction in the dystrophin-glycoprotein complex and secondary alterations in the membrane-associated muscle proteome. The mass spectrometry-based proteomic analysis of the crude microsomal fraction obtained from six-month old hind leg muscle from *mdx-4cv* mice *versus* age-matched wild type mice revealed new protein changes in a relatively unexplored mouse model of X-linked muscular dystrophy. This included the identification of key members of the dystrophin-glycoprotein complex and various low copy number proteins. Alterations in protein expression were identified by a highly reliable label-free LC-MS/MS approach and significant changes in muscle-associated proteins of interest were verified by immunoblot analysis. Increases in the Na^+^/K^+^-ATPase were also confirmed by immunofluorescence microscopy.

### 3.1. Organelle Proteomic Analysis of Skeletal Muscle

**Figure 1:**
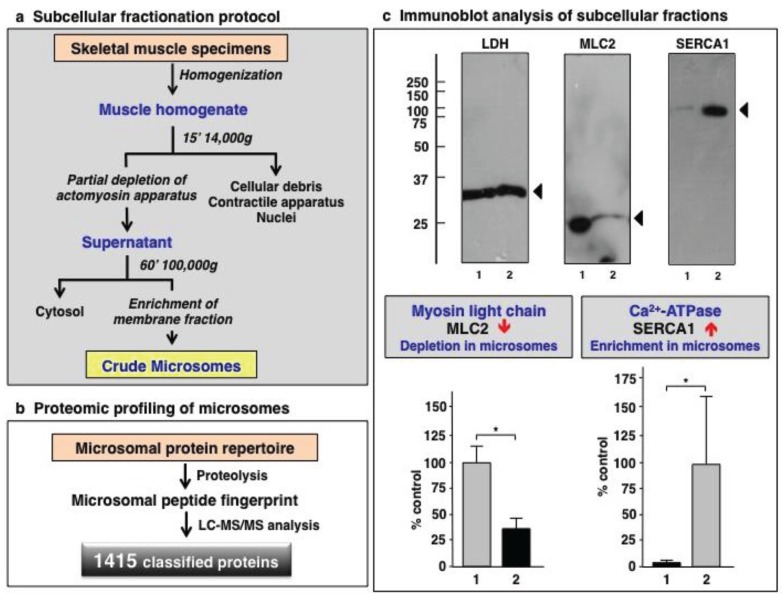
(**a**) Flowchart of the subcellular fractionation approach used in this proteomic study for the isolation of crude microsomes from skeletal muscle. (**b**) Overview of the mass spectrometric profiling of the microsomal subproteome. (**c**) Immunoblot analysis of lactate dehydrogenase (LDH; as loading control), myosin light chain isoform MLC2 and the sarcoplasmic reticulum Ca^2+^-ATPase isoform SERCA1. Arrowheads indicate immuno-labelled bands. Lanes 1 and 2 represent total muscle extracts and the microsomal fraction, respectively. Graphical presentations show the statistical analysis of immunoblotting of the contractile apparatus marker MLC2 *versus* the membrane fraction marker SERCA1 (Student’s *t*-test, unpaired; *n* = 4; * *p* < 0.05).

This study aimed to investigate the suitability of a simple differential centrifugation protocol for the removal of a large portion of the contractile apparatus from the muscle homogenate prior to label-free mass spectrometric analysis. This type of organelle biochemistry usually enables the exploration of low abundance proteins in the hidden proteome [20,21,22], which may otherwise be masked by the highly abundant actomyosin assembly in conventional studies of crude muscle homogenates [19]. Since low copy number proteins may represent important biomarkers of muscular dystrophy [68], the systematic profiling of the deep proteome may improve our detailed understanding of its pathogenesis [69]. The success of this subcellular fractionation technique was evaluated by gel electrophoresis and immunoblotting. The results obtained confirm the removal of a considerable part of the contractile apparatus, leaving behind a microsomal pellet enriched in many membrane-associated protein species. Figure 1 outlines the subcellular fractionation protocol, the proteomic profiling approach and the immunoblot analysis of established protein markers of the contractile apparatus *versus* the membrane fraction from skeletal muscle. While the MLC2 isoform of myosin light chain [70] was shown to be greatly reduced in the microsomal fraction, the concentration of the fast SERCA1 isoform of the sarcoplasmic reticulum Ca^2+^-ATPase [71] was clearly increased following differential centrifugation. Hence, immunoblotting clearly demonstrated the partial depletion of the actomyosin apparatus and concomitant enrichment of membrane-associated proteins in the microsomal fraction from skeletal muscle homogenates.

**Figure 2:**
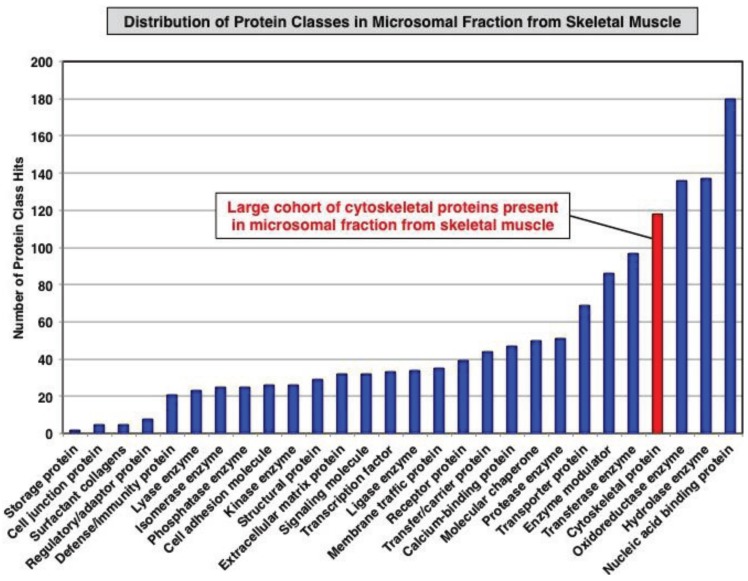
Overview of the distribution of protein classes present in the microsomal fraction from skeletal muscle as judged by label-free LC-MS/MS analysis and the bioinformatics listing according to the PANTHER database of protein families for the cataloguing of molecular functions [58].

The proteomic classification of the entire microsomal protein repertoire identified 1415 distinct molecular species. As summarized in Figure 2, these proteins fall into a variety of functional classes, such as cell junction proteins, surfactant collagens, regulatory/adaptor proteins, defence/immunity proteins, lyases, isomerases, phosphatases, cell adhesion molecules, kinases structural proteins, extracellular matrix proteins, signalling molecules, transcription factors, ligases, membrane traffic proteins, receptor proteins, transfer/carrier proteins, Ca^2+^-binding proteins, molecular chaperones, proteases, transporter proteins, enzyme modulators, transferases, cytoskeletal proteins, oxidoreductases, hydrolases and nucleic acid binding proteins. Crucially, a considerable number of cytoskeletal proteins were identified in the microsomal fraction, which makes this membrane-enriched fraction highly suitable for the systematic and simultaneous screening of the molecular fate of the dystrophin-glycoproteins complex and the skeletal muscle subproteome consisting of low abundance components and integral proteins.

### 3.2. Label-Free LC-MS/MS Analysis of mdx-4cv *versus* Wild Type Skeletal Muscle Microsomes

**Figure 3:**
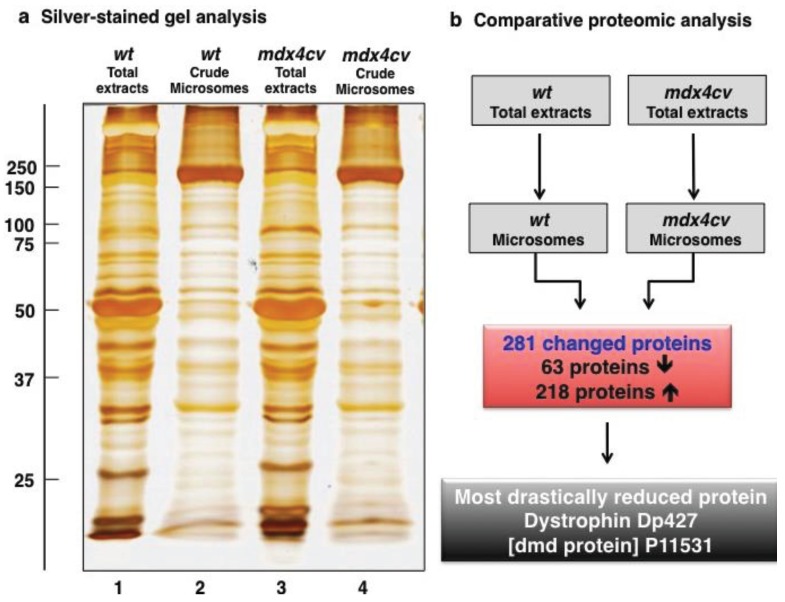
(**a**) Gel electrophoretic analysis of total muscle extracts (lanes 1 and 3) and the crude microsomal muscle fraction (lanes 2 and 4) from wild type (*wt*; lanes 1 and 2) *versus* dystrophic *mdx-4cv* (lanes 3 and 4) mice. Molecular mass standards (in kDa) are marked to the left of the panel. (**b**) Overview of the findings from the comparative proteomic analysis of the microsomal fraction from dystrophic *versus* wild type muscle.

The successful application of differential centrifugation for separating abundant contractile proteins from the microsomal fraction was substantiated by gel electrophoretic analysis. As illustrated in Figure 3, the total tissue extract and the microsomal fraction exhibited clearly different protein banding patterns in silver-stained 1D gels. A label-free LC-MS/MS analysis was conducted with six-month old *mdx-4cv* samples *versus* age-matched leg muscle microsomes to investigate differential protein expression patterns between dystrophic and normal skeletal muscle. The proteomic profiling of the microsomal fraction revealed altered concentration levels of 281 proteins in the dystrophic *mdx-4cv* mouse model. An increased abundance was detected for 218 proteins and a decrease was found for 63 proteins.

Importantly, the most drastically reduced protein in the microsomal fraction from *mdx-4cv* skeletal muscle was identified as the full-length isoform Dp427 of dystrophin (P11531) [34]. As listed in Table 1 and Table S1 (supplementary material), a variety of components of the dystrophin-associated glycoprotein complex were also clearly identified as being reduced in the *mdx-4cv* microsomal fraction, including dystroglycan (Q62165), δ-sarcoglycan (P82347), γ-sarcoglycan (P82348) and α1-syntrophin (Q61234) [46]. This is a crucial result as for the first time the primary cause of muscular dystrophy, *i.e.*, the absence of dystrophin and resulting decrease in components of its associated glycoprotein complex, could be determined along downstream abnormalities within the one sample and using a single analytical run. Previous biochemical studies demonstrated the significant reduction of the dystrophin-glycoprotein complex in both *mdx* skeletal muscles [72] and biopsy specimens from patients afflicted with Duchenne muscular dystrophy [73]. These primary pathobiochemical changes within the cytoskeletal network could now be directly related to potential downstream effects on other cellular mechanisms, functions and structures. Rearrangements in the actomyosin apparatus were reflected by reduced levels of certain myosins and actins, as well as regulatory proteins of the sarcomeric structure. In addition, a variety of metabolic enzymes and metabolite transporters of both anaerobic and oxidative pathways were shown to be of lower concentration in dystrophin-deficient muscle preparations. The analysis of the *mdx-4cv* microsomal fraction clearly confirmed a reduced abundance of myozenin MYZ-1 (Q9JK37), carbonic anhydrase isoform CA3 (P16015), myoglobin (P04247) the fast isoform of myosin-binding protein MBP-C (Q5XKE0) and myomesin (Q62234) in dystrophin-deficient muscle tissue. This agrees with a variety of previously published proteomic studies, as critically examined in a recent review on the proteomics of the dystrophin-glycoprotein complex and dystrophinopathy [46].

**Table 1:** List of changed muscle proteins with a reduced concentration of more than 3-fold in the *mdx-4cv* microsomal fraction (Table S1 in the supplementary materials lists additional proteomic hits).

Accession No.	Protein Name	Peptide Count	Confidence Score	Anova (*p*)	Fold Change
P11531	Dystrophin Dp427	15	744.3	5.95E-05	–16.01
Q62165	Dystroglycan	9	396.3	4.50E-06	–9.17
Q6P3A8	2-oxoisovalerate dehydrogenase subunit beta, mitochondrial	2	172.9	0.007920	–7.24
P68040	Guanine nucleotide-binding protein subunit beta-2-like 1	2	113.4	0.007929	–6.53
P51667	Myosin regulatory light chain 2, ventricular/cardiac muscle isoform	3	167.4	0.037556	–6.52
P62737	Actin, aortic smooth muscle	7	407.6	0.008389	–6.19
P50136	2-oxoisovalerate dehydrogenase subunit alpha, mitochondrial	7	360.6	8.15E-05	–5.06
Q9Z2I8	Succinyl-CoA ligase (GDP-forming) subunit beta, mitochondrial	2	65.4	0.005705	–4.98
P09542	Myosin light chain 3	7	435.9	0.021966	–4.68
P82348	Gamma-sarcoglycan	3	139.3	0.013191	–4.08
P53395	Lipoamide acyltransferase of branched alpha-keto acid dehydrogenase	12	531.9	3.36E-05	–3.97
P68134	Actin, alpha skeletal muscle	2	67.6	0.019843	–3.85
Q9WUB3	Glycogen phosphorylase, muscle form	4	58.3	0.006224	–3.85
P70695	Fructose-1,6-bisphosphatase isozyme 2	10	604.8	0.002383	–3.83
Q9CPP6	NADH dehydrogenase [ubiquinone] 1 alpha subcomplex subunit 5	2	48.3	0.027033	–3.74
Q91YE8	Synaptopodin-2	4	368.3	0.009175	–3.70
Q8K370	Acyl-CoA dehydrogenase family member 10	11	414.0	0.000992	–3.57
Q9JI91	Alpha-actinin-2	8	493.8	0.034239	–3.43
Q9JK37	Myozenin-1	5	308.7	0.029472	–3.41
O88990	Alpha-actinin-3	59	5419.6	0.008821	–3.37
P16015	Carbonic anhydrase 3	22	1821.9	2.98E-05	–3.31
O88492	Perilipin-4	2	130.3	1.58E-05	–3.29
Q8BVI4	Dihydropteridine reductase	3	206.2	0.000804	–3.28
P63323	40S ribosomal protein S12	2	116.9	0.027770	–3.22
Q91ZJ5	UTP-glucose-1-phosphate uridylyltransferase	20	1265.2	2.53E-05	–3.16
Q7TPR4	Alpha-actinin-1	17	1205.8	0.011164	–3.11

A very large number of muscle-associated proteins were found to be increased in the *mdx-4cv* animal model of X-linked muscular dystrophy, as listed in Table 2 and Table S2 (supplementary material). This included proteins involved in cell regulation, muscle contraction, metabolite transportation, energy metabolism, maintenance of the extracellular matrix, cytoskeletal network formation, and the cellular stress response. Some of these proteome-wide changes may be compensatory mechanisms to protect the weakened surface membrane system and strengthen the instable cytoskeleton, or reflect secondary pathological changes such as myofibrosis or metabolic disturbances. A select group of these altered proteins may be suitable as indicator candidates for the establishment of a comprehensive and muscle-associated biomarker signature of Duchenne muscular dystrophy [68,69]. The most highly increased protein in dystrophin-deficient hind limb muscle was identified as alpha-1-antitrypsin (Q00898) [74], a major anti-protease, which is probably a multi-functional type of protein [75]. Besides being involved in diminishing the destructive effects of major proteases, alpha-1-antitrypsin exhibits also anti-inflammatory properties [76]. Hence, its up-regulation in muscular dystrophy could be interpreted as both an anti-protease and an anti-inflammatory response. High levels of the matricellular protein periostin (Q62009) agree with remodelling of the extracellular matrix [77]. Another characteristic feature of muscular dystrophy-associated fibrosis appears to be an increase in fibrinogen (Q8VCM7, Q8KOE8). A previous proteomic study had already established an up-regulation of fibrinogen in the aged *mdx* diaphragm [43] and this was confirmed here to also occur in *mdx-4cv* hind limb muscle. Increased fibrinogen was shown to affect the TGF-β/alternative macrophage activation pathway in dystrophin-deficient muscle and therefore plays a crucial role in fibrosis [78].

**Table 2:** List of changed proteins with an increased concentration of more than 5-fold in the *mdx-4cv* microsomal fraction (Table S2 in the supplementary materials lists additional proteomic hits).

Accession No.	Protein Name	Peptide Count	Confidence Score	Anova (*p*)	Fold Change
Q00898	Alpha-1-antitrypsin 1–5	6	607.1	2.05E-05	158.43
Q61941	NAD(P) transhydrogenase, mitochondrial	22	1020.3	0.001141	62.16
Q8C013	Trophoblast glycoprotein-like	2	126.07	0.010285	52.42
Q91WC3	Long-chain-fatty-acid-CoA ligase 6	2	67.7	0.012257	32.60
O70152	Dolichol-phosphate mannosyltransferase	2	47.5	0.000369	20.15
Q61233	Plastin-2	3	145.4	0.000393	18.30
P41317	Mannose-binding protein C	6	527.3	1.80E-06	16.38
Q62351	Transferrin receptor protein 1	6	264.2	0.003053	16.02
P16110	Galectin-3	6	553.4	1.05E-06	15.93
Q9D154	Leukocyte elastase inhibitor A	8	446.3	1.45E-06	15.48
P09541	Myosin light chain 4	2	121.1	0.000221	13.67
Q61878	Bone marrow proteoglycan	2	80.3	0.000859	13.43
Q69ZN7	Myoferlin	2	91.1	0.000864	12.72
P14434	H-2 class II histocompatibility antigen, A–B alpha chain	3	109.3	0.000268	11.95
Q924X2	Carnitine O-palmitoyltransferase 1, muscle isoform	2	66.4	0.032150	10.69
Q62009	Periostin	4	156.5	0.000967	9.92
Q8VCM7	Fibrinogen gamma chain	3	172.5	5.79E-05	9.15
Q9DBS1	Transmembrane protein 43	7	428.6	0.002721	8.61
Q9CQW9	Interferon-induced transmembrane protein 3	2	55.2	0.002680	8.44
Q6PD26	GPI transamidase component PIG-S	4	158.2	0.013246	8.15
Q9DC16	Endoplasmic reticulum-Golgi intermediate compartment protein 1 E	2	134.5	0.016933	7.76
P28665	Murinoglobulin-1	11	522.1	0.000139	7.51
P05555	Integrin alpha-M	6	230.6	0.000560	7.39
P09470	Angiotensin-converting enzyme	10	450.2	0.001196	7.28
P14483	H-2 class II histocompatibility antigen, A beta chain	2	9.3	0.001178	7.13
Q68FD5	Clathrin heavy chain 1	4	158.9	0.012682	7.01
Q9R069	Basal cell adhesion molecule	4	131.2	0.000134	6.98
Q06890	Clusterin	6	271.7	0.003051	6.97
P61620	Protein transport protein Sec61 subunit alpha isoform 1	2	115.9	0.032657	6.80
P01898	H-2 class I histocompatibility antigen, Q10 alpha chain	2	45.0	0.000994	6.47
Q6IRU2	Tropomyosin alpha-4 chain	2	59.5	0.001981	6.39
Q8BMD8	Calcium-binding mitochondrial carrier protein SCaMC-1	2	94.8	0.018072	6.30
Q8K0E8	Fibrinogen beta chain	4	221.8	9.94E-05	6.28
Q8BMK4	Cytoskeleton-associated protein 4	8	529.1	0.002108	6.27
Q9WUQ2	Prolactin regulatory element-binding protein	2	45.5	0.024108	6.23
P11835	Integrin beta-2	4	327.7	0.002931	6.22
Q9QZF2	Glypican-1	6	405.6	0.001895	5.96
Q8C129	Leucyl-cystinyl aminopeptidase	5	240.9	0.009879	5.95
P49290	Eosinophil peroxidase	11	581.4	0.010892	5.67
Q9D7J6	Deoxyribonuclease-1-like 1	2	145.7	0.029576	5.65
Q9ERD7	Tubulin beta-3 chain	2	96.3	0.002934	5.57
P14426	H-2 class I histocompatibility antigen, D–K alpha chain	2	156.2	0.002441	5.47
P57716	Nicastrin	2	114.6	0.006051	5.43
Q61704	Inter-alpha-trypsin inhibitor heavy chain H3	2	123.4	0.011644	5.37
Q60854	Serpin B6	11	601.5	1.68E-06	5.07

The considerable increase in tubulin (Q9ERD7; Q9D6F9; P99024), vinculin (Q64727) and vimentin (P20152) indicated a restructuring of the dystrophin-lacking cytoskeletal network system in dystrophic fibres [46]. Changed annexin isoforms A1, A2, A4, A5 and A6 (P10107; P07356; P97429; P48036; Q07076) levels strongly implied abnormalities in Ca^2+^-binding and overall Ca^2+^-homeostasis, as well as altered membrane organization [79]. Annexin A1 is closely linked to the maintenance of the muscle cytoskeleton and also extracellular matrix integrity [80] and its increased concentration might therefore represent a compensatory mechanism to stabilise the dystrophin-deficient fibre surface. A very interesting new proteomic finding of this label-free approach was the significant increase in a major integral membrane protein of the sarcolemma, the ouabain-sensitive Na^+^/K^+^-ATPase. Damaged skeletal muscle fibres appear to compensate abnormal ion fluxes by increasing the availability of this ion pumping protein in order to stabilise the membrane potential over the dystrophin-lacking plasmalemma [81]. The fact that this proteomic analysis was based on liquid chromatography and therefore enabled the detailed analysis of integral proteins, shows that disadvantages of purely gel-based methods can be partially overcome by using alternative protein separation techniques [19,20,21,22,23].

### 3.3. Bioinformatics Evaluation of the Label-Free LC-MS/MS Analysis of mdx-4cv Muscle Microsomes

The bioinformatics PANTHER database of protein families [58] was used to identify the molecular functions of the newly identified *mdx-4cv* muscle proteins with an altered abundance in the microsomal fraction. For the microsomal subproteome, the proposed molecular functions were established as follows: Antioxidant activity (0.7%), binding activity (23.2%), catalytic activity (37.2%), enzyme regulator activity (7.8%), nucleic acid binding transcription factor activity (1.4%), protein binding transcription factor activity (0.3%), receptor activity (6.5%), structural molecule activity (15.4%), and transporter activity (7.5%).

**Figure 4:**
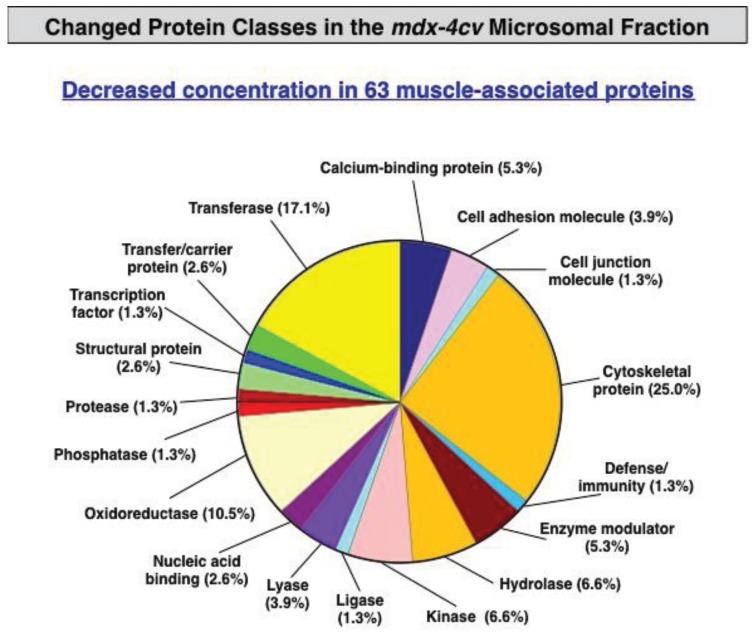
Summary of changed protein classes with a reduced abundance in *mdx-4cv* hind limb muscle. The bioinformatics software programme PANTHER [57,58] was applied to identify the clustering of protein classes based on the mass spectrometric analysis of the microsomal fraction from dystrophic *versus* control specimens (Table 1 and Table S1).

**Figure 5:**
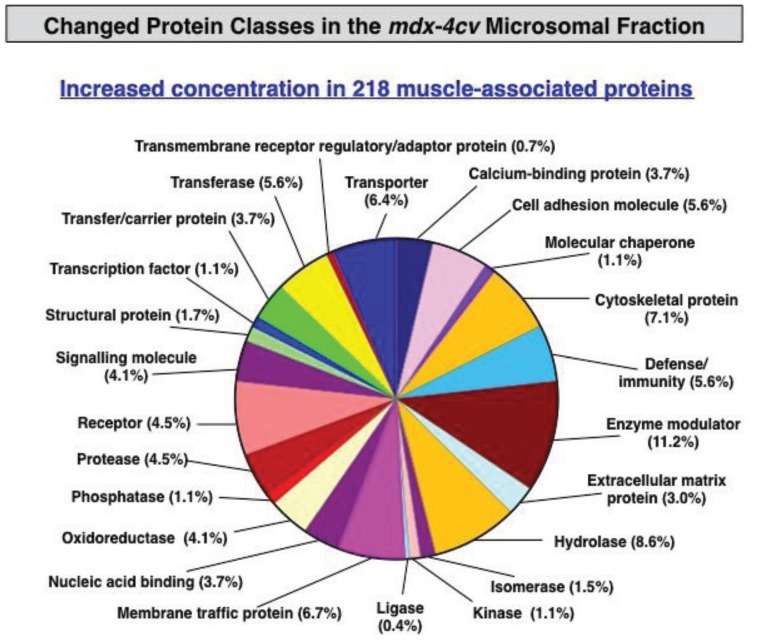
Summary of changed protein classes with an increased abundance in *mdx-4cv* hind limb muscle. The bioinformatics software programme PANTHER [57,58] was applied to identify the clustering of protein classes based on the mass spectrometric analysis of the microsomal fraction from dystrophic *versus* control specimens (Table 2 and Table S2).

Changes in protein classes are presented in Figure 4 and Figure 5, illustrating the large diversity of altered types of protein in muscular dystrophy. Figure 4 displays the distribution of decreased protein classes and Figure 5 shows the increased muscle protein families. The class of cytoskeletal proteins was estimated to cover approximately 11% of the total cohort of changed proteins. Remarkably, a quarter of decreased components were shown to be cytoskeletal proteins (Figure 4), agreeing with the fact that dystrophinopathies are primary diseases of the membrane cytoskeleton. Thus, the deficiency in the full-length dystrophin isoform Dp427 has a significant effect on the entire cytoskeletal network of dystrophic muscle fibres.

The bioinformatics database STRING for determining potential protein-protein interaction networks [59,60] was used to evaluate the patterns of change in direct physical and indirect functional protein interactions. The analysis of mass spectrometrically identified proteins with a changed abundance in *mdx-4cv* muscle was based on the proteomic data presented in Table 1, Table 2, Table S1 and Table S2. The analysis clearly illustrated the central position of the altered dystrophin-dystroglycan axis and its involvement in muscular dystrophy, as well as the changed interaction patterns between other elements of the cytoskeletal network and the contractile apparatus (Figure S1; supplementary materials).

### 3.4. Verification Analysis of Increased Levels of the Na^+^/K^+^-ATPase in mdx-4cv Skeletal Muscle

**Figure 6:**
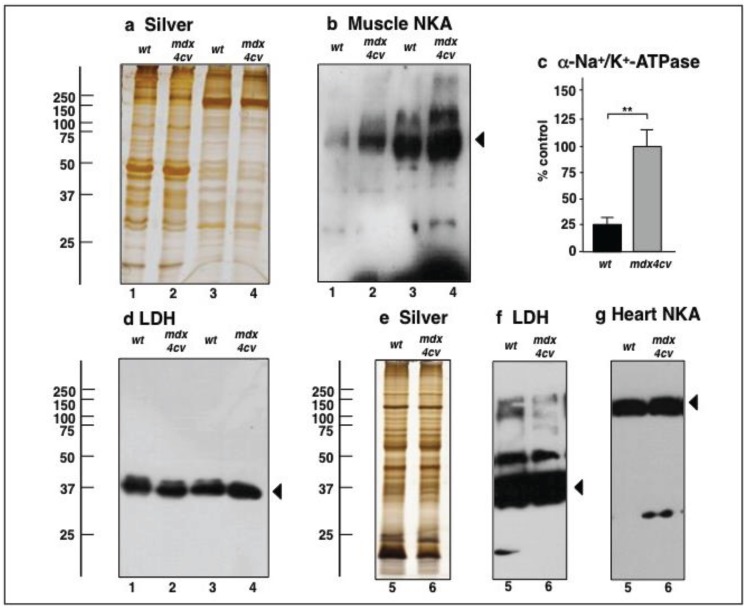
Immunoblot analysis of the Na^+^/K^+^-ATPase from *mdx-4cv* skeletal and cardiac muscles. Shown are representative silver-stained gels (**a**,**e**) and immunoblots (**b**,**d**,**f**,**g**). Lanes 1 to 6 represent total extracts from non-dystrophic wild type (*wt*) skeletal muscle, total extracts from dystrophic *mdx-4cv* skeletal muscle, crude microsomes from *wt* skeletal muscle, crude microsomes form *mdx-4cv* skeletal muscle, total extracts from *wt* heart, and total extracts from *mdx-4cv* heart, respectively. Blots were labelled with antibodies to the Na^+^/K^+^-ATPase (**b**,**g**), and lactate dehydrogenase (LDH; as a loading control) (**d**,**f**). Arrowheads indicate immuno-labelled bands. Graphical representations of the immuno-decoration levels for the Na^+^/K^+^-ATPase in normal *versus* *mdx-4cv* microsomes are shown in panel (**c**): Student’s *t*-test, unpaired; *n* = 4; ** *p* < 0.01. See supplementary Figure S2 for the graphical presentation of LDH and the cardiac Na^+^/K^+^-ATPase.

**Figure 7:**
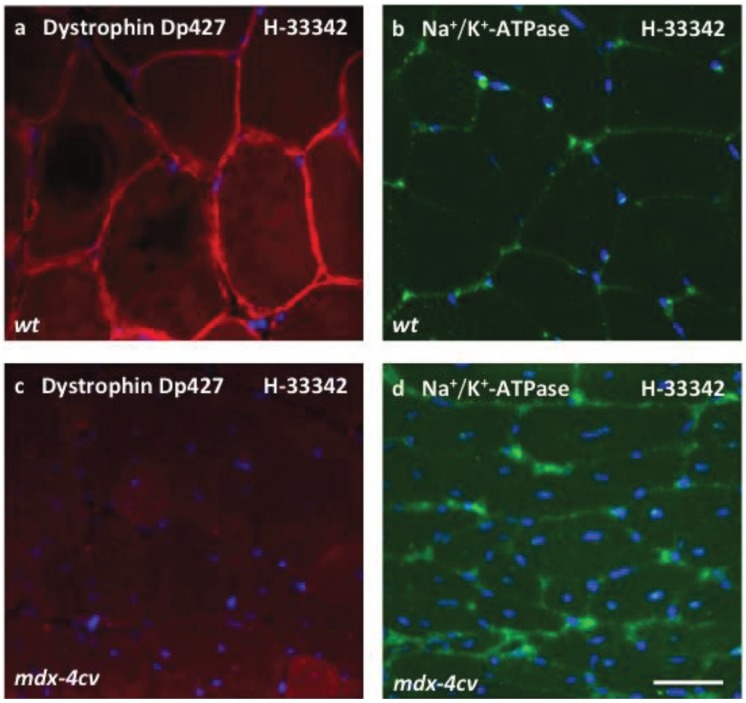
Immunofluorescence microscopical analysis of dystrophin and the Na^+^/K^+^-ATPase in *mdx-4cv* *gastrocnemius* muscle. Shown is the labelling of nuclei with the DNA binding dye bis-benzimide Hoechst 33342 (H-33342) (**a**–**d**), as well as antibody labelling of the full-length dystrophin isoform Dp427 (**a**,**c**) and the Na^+^/K^+^-ATPase (**b**,**d**) in wild type (*wt*) (**a**,**b**) *versus* dystrophic *mdx-4cv* (**c**,**d**) *gastrocnemius* muscle. The immunofluorescence microscopical analysis of dystrophin-deficient muscle fibres indicates a higher degree of central nucleation and increased levels of the sarcolemmal Na^+^/K^+^-ATPase. The bar equals 20 μm.

To demonstrate that the results from organelle proteomics reflect changes in the entire skeletal muscle proteome, immunoblotting was carried out with both total extracts and the microsomal fraction for the evaluation of a major integral membrane protein, the α-subunit of the Na^+^/K^+^-ATPase. In agreement with previous biochemical and physiological studies [81], this sarcolemmal protein was shown to be significantly increased in both total tissue extracts and the microsomal fraction from dystrophic skeletal muscle (Figure 6b,c). Immuno-labelling of lactate dehydrogenase was employed as a loading control (Figure 6d). This metabolic enzyme is relatively abundant in muscle tissues and was not identified as being changed in the microsomal fraction from *mdx-4cv* muscles. It was therefore employed as a convenient loading control in our immunoblotting survey of changed proteins. Importantly, the comparative immunoblot analysis of the Na^+^/K^+^-ATPase in normal *versus* dystrophic cardiac muscle showed no major differences in the density of this ion-pumping protein in the *mdx-4cv* heart (Figure 6g; supplementary Figure S2). Hence, the apparent up-regulation of the Na^+^/K^+^-ATPase appears to be restricted to dystrophic skeletal muscles of the investigated age group of *mdx-4cv* mice. Findings form immunofluorescence microscopy agreed with both the proteomic survey and the immunoblot analysis presented in this report. As shown in Figure 7, dystrophin-deficient muscle fibres exhibited an increased abundance of the Na^+^/K^+^-ATPase in the sarcolemma membrane and also a higher degree of central nucleation. Thus, the increased levels of the Na^+^/K^+^-ATPase in the *mdx-4cv* preparations verify the proteomic findings listed in Table S2 (supplementary materials) and also confirm that label-free mass spectrometry is highly suitable for the comparative analysis of integral membrane proteins from skeletal muscles.

### 3.5. Immunoblotting Survey of mdx-4cv *versus* Wild Type Skeletal Muscle

**Figure 8:**
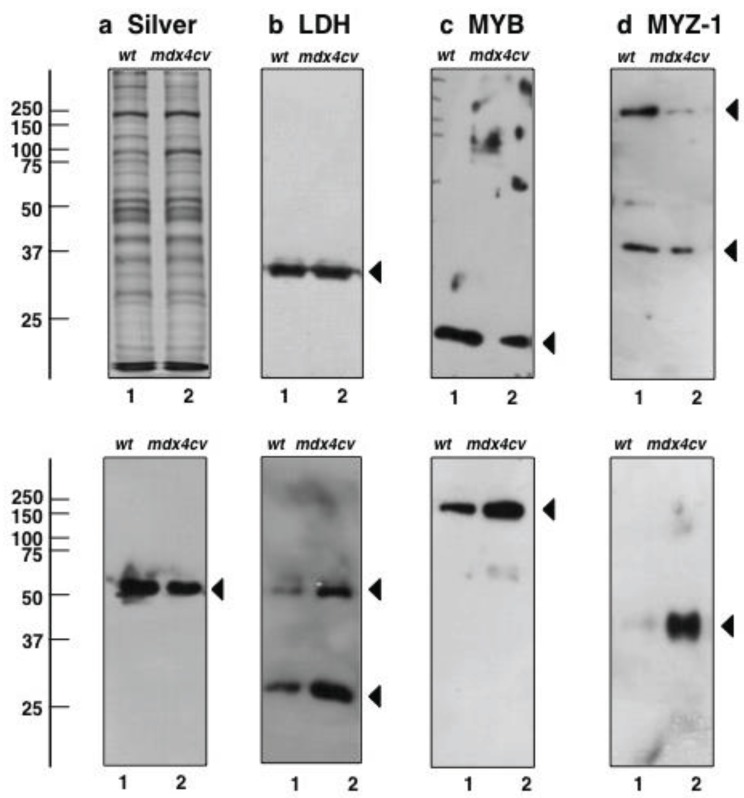
Immunoblot analysis of proteins with a considerable change in concentration levels in *mdx-4cv* skeletal muscle tissue. Shown is a silver-stained gel (**a**) and representative immunoblots (**b**–**h**). Lanes 1 and 2 represent crude microsomes from wild type muscle and dystrophic *mdx-4cv* muscle, respectively. Blots were labeled with antibodies to lactate dehydrogenase (LDH) (**b**), myoglobin (MYB) (**c**), myozenin (MYZ-1) (**d**), pyruvate dehydrogenase (PDH) (**e**), β-tubulin (β-TUB) (f), periostin (POSTN) (**g**) and vimentin (VIM) (**h**). The graphical representations of immuno-decoration levels are shown in supplementary Figure S3. Arrowheads indicate immuno-labelled bands. In the case of myozenin and tubulin, high-molecular-mass bands were detected besides the main isoforms, possibly representing larger protein aggregates.

Select findings from the proteomic survey of the microsomal fraction from *mdx-4cv* hind limb muscle were verified by comparative immunoblot analysis, so this new proteomic survey could be related to previous large-scale analyses of muscles from dystrophic mice. This included established marker proteins of decreased and increased abundance in muscular dystrophy. Silver-stained gels showed no major changes in the overall protein band pattern in wild type *versus* dystrophic muscle microsomes following gel electrophoretic separation (Figure 8a). Antibody labelling of lactate dehydrogenase was used as a loading control (Figure 8b). The immunoblotting of myoglobin, a major muscle-associated oxygen carrier, myozenin isoform 1, an intracellular binding protein that provides a linkage of the Z-line proteins α-actinin and γ-filamin to the sarcomere structure, and pyruvate dehydrogenase, a major mitochondrial protein involved in the conversion of pyruvate into acetyl-CoA, indicated a reduction in *mdx-4cv* skeletal muscle (Figure 8c–e). This agrees with previous proteomic studies, as reviewed by Holland *et al.* [46] and Dowling *et al.* [69]. In contrast, the microtubular element β-tubulin, the matricellular protein periostin and the intermediate filament component vimentin were found to be significantly increased in dystrophic muscles (Figure 8f–h; supplementary Figure S3), which is also in agreement with a previous study [77].

## 4. Conclusions

In order to establish a large number of predictive, diagnostic, prognostic and/or therapy monitoring biomarkers in relation to dystrophinopathy-associated changes, international research initiatives have focused on the large-scale and high-throughput screening of dystrophic muscle specimens. In analogy to these systematic efforts, we present here the first proteomic documentation of the actual loss of dystrophin and the significant decrease of dystrophin-associated proteins in skeletal muscle preparations. Previous proteomic analyses of total muscle tissue extracts employing a single analytical run have failed to detect these changes that present the primary defect in X-linked muscular dystrophy. In this new analytical approach, the comprehensive analysis of the crude microsomal fraction allowed the simultaneous proteomic evaluation of both the primary abnormality of dystrophinopathy plus downstream alterations within the same muscle sample. Secondary alterations included a large number of proteins involved in cell signalling, energy metabolism, the excitation-contraction-relaxation cycle, ion homeostasis, the cellular stress response, the extracellular matrix and the cytoskeletal network. It is important to emphasise that the interpretation of comparative findings from proteomic studies of crude extracts or distinct subcellular fractions from diseased tissues is complicated and potentially adversely affected by the dissimilar cellular composition of analysed specimens. The dystrophy-related substitution of degenerating contractile fibres by fatty and connective tissue therefore presents an issue when one tries to interpret proteomic changes in wild type *versus* dystrophic phenotypes. However, in the case of the dystrophic hind leg muscle investigated in this study, relatively low levels of fibrosis have been observed, as compared to the *mdx* diaphragm or the aging dystrophic heart muscle. Therefore, the findings from our organelle proteomic survey of a moderately affected group of skeletal muscles should be relatively representative of actual changes within the muscle proteome. Importantly, the usefulness of the label-free LC-MS/MS technology for the improved proteomic coverage of certain classes of proteins, which are usually under-represented in gel-based studies, was exemplified by the identification of hydrophobic protein species such as the sarcolemmal Na^+^/K^+^-ATPase. Thus, ideally both gel-free and gel-based methods should be combined to identify the maximum number of diverse biomarker candidates with greatly differing physicochemical properties.

## Supplementary Files

Supplementary File 1
