# Supplementary material for: Simultaneous Pathoproteomic Evaluation of the Dystrophin-Glycoprotein Complex and Secondary Changes in the mdx-4cv Mouse Model of Duchenne Muscular Dystrophy
Source: Biology (Basel). 2015 Jun 10;4(2):397–423. doi: 10.3390/biology4020397 (PMC4498307; doi:10.3390/biology4020397)
Supplement: Supplementary File 1 [file biology-04-00397-s001.pdf]

Supplementary Materials

Table S1. List of changed proteins with a reduced concentration of less than 3-fold in *mdx-4cv* microsomes.

Accession No.	Protein Name	Peptide Count	Confidence Score	Anova (*p*)	Fold Change
P15626	Glutathione S-transferase Mu 2	2	130.8	0.004045	−2.92
P17563	Selenium-binding protein 1	6	336.2	0.001289	−2.92
Q99LC5	Electron transfer flavoprotein subunit alpha, mitochondrial	2	71.5	0.002063	−2.92
P70402	Myosin-binding protein H	7	405.8	0.013635	−2.90
P47857	6-phosphofructokinase, muscle type	8	588.7	0.020360	−2.84
Q8C0M9	Isoaspartyl peptidase/L-asparaginase	4	206.7	0.000256	−2.83
P04247	Myoglobin	12	1654.9	0.000525	−2.82
Q8VCT4	Carboxylesterase 1D	8	421.1	0.005918	−2.81
P06801	NADP-dependent malic enzyme	6	194.7	0.000931	−2.77
P42669	Transcriptional activator protein Pur-alpha	5	406.5	0.001367	−2.72
P82347	Delta-sarcoglycan	3	255.1	0.033834	−2.59
O55103	Periaxin	23	1163.1	0.010324	−2.58
Q61234	Alpha-1-syntrophin	4	102.7	0.008697	−2.58
Q5XKE0	Myosin-binding protein C, fast-type	3	54.0	0.027034	−2.53
P70195	Proteasome subunit beta type-7	3	244.3	0.003034	−2.51
Q62234	Myomesin-1	72	4983.3	0.001161	−2.49
Q05920	Pyruvate carboxylase, mitochondrial	20	1047.0	0.003850	−2.48
P58771	Tropomyosin alpha-1 chain	3	93.2	0.028163	−2.48
Q9EQ20	Methylmalonate-semialdehyde dehydrogenase, mitochondrial	2	119.3	0.016796	−2.46
P05977	Myosin light chain 1/3, skeletal muscle isoform	2	127.0	0.044906	−2.41
Q9QZ47	Troponin T, fast skeletal muscle	2	277.0	0.004359	−2.37
Q8BKZ9	Pyruvate dehydrogenase protein X component, mitochondrial	19	1589.9	0.000349	−2.34
Q9DAK9	14 kDa phosphohistidine phosphatase	4	316.3	0.000121	−2.31
Q8R1G2	Carboxymethylenebutenolidase homolog	3	266.2	0.025153	−2.31
P04370	Myelin basic protein	4	223.5	0.014370	−2.30
P09405	Nucleolin	12	717.6	0.001197	−2.30
Q8VCR8	Myosin light chain kinase 2, skeletal/cardiac muscle	3	120.9	5.90E-05	−2.27
P54775	26S protease regulatory subunit 6B	13	746.0	0.000148	−2.25
Q99MN9	Propionyl-CoA carboxylase beta chain, mitochondrial	9	421.8	0.007876	−2.18
Q60675	Laminin subunit alpha-2	4	178.3	0.001218	−2.17
P46471	26S protease regulatory subunit 7	7	392.4	0.008889	−2.12
P62204	Calmodulin	4	402.5	0.007982	−2.11
P26443	Glutamate dehydrogenase 1, mitochondrial	3	196.8	8.78E-05	−2.07
Q6P8J7	Creatine kinase S-type, mitochondrial	4	263.7	0.013513	−2.06
Q5SX39	Myosin-4	5	274.5	0.027764	−2.04
O54962	Barrier-to-autointegration factor	4	259.5	0.003174	−2.04
Q8BG32	26S proteasome non-ATPase regulatory subunit 11	2	90.1	0.017666	−2.03

Table S2. List of changed proteins with an increased concentration of less than 5-fold in *mdx-4cv* microsomes.

Accession No.	Protein Name	Peptide Count	Confidence Score	Anova (*p*)	Fold Change
P08752	Guanine nucleotide-binding protein G(i) subunit alpha-2	5	197.1	0.003593	4.95
P49300	C-type lectin domain family 10 member A	3	356.8	2.08E-05	4.89
P84078	ADP-ribosylation factor 1	7	132.4	0.002455	4.83
Q9D6F9	Tubulin beta-4A chain	2	43.9	0.003541	4.82
Q8R5J9	PRA1 family protein 3	5	421.6	0.004263	4.81
Q8BY89	Choline transporter-like protein 2	4	147.9	0.030126	4.80
Q9DCN2	NADH-cytochrome b5 reductase 3	8	832.3	0.002056	4.68
Q8BLF1	Neutral cholesterol ester hydrolase 1	3	180.1	0.014434	4.63
P26039	Talin-1	5	250.3	0.000536	4.51
Q9CPU4	Microsomal glutathione S-transferase 3	4	187.2	0.033598	4.47
Q91V61	Sideroflexin-3	7	448.4	0.002239	4.43
Q08857	Platelet glycoprotein 4	3	133.6	0.037377	4.39
P08226	Apolipoprotein E	6	404.5	1.98E-05	4.39
Q9Z0M6	CD97 antigen	4	215.9	0.000687	4.38
O88587	Catechol O-methyltransferase	3	206.3	0.026071	4.33
Q3TDQ1	Dolichyl-diphosphooligosaccharide protein glycosyltransferase SU-STT3B	4	213.4	0.006603	4.32
B2RXS4	Plexin-B2	3	96.5	0.000328	4.31
Q61735	Leukocyte surface antigen CD47	2	131.1	0.007560	4.30
P51881	ADP/ATP translocase 2	5	177.1	0.000472	4.25
O35643	AP-1 complex subunit beta-1	2	141.3	0.002891	4.19
Q3TZZ7	Extended synaptotagmin-2	4	302.8	0.023359	4.14
P01899	H-2 class I histocompatibility antigen, D-B alpha chain	5	188.1	0.000257	4.04
Q9ESD7	Dysferlin	15	780.6	0.029892	4.02
Q3U7R1	Extended synaptotagmin-1	10	546.4	0.005081	4.01
P49817	Caveolin-1	4	159.9	0.024592	3.98
P10833	Ras-related protein R-Ras	2	51.8	0.015348	3.97
Q61738	Integrin alpha-7	13	808.7	0.004887	3.97
P40124	Adenylyl cyclase-associated protein 1	2	233.2	0.004418	3.97
P06909	Complement factor H	4	124.0	0.010462	3.97
Q9D1G3	Protein-cysteine N-palmitoyltransferase HHAT-like protein	2	123.9	0.038319	3.81
P27659	60S ribosomal protein L3	5	222.6	0.001766	3.80
Q8C7X2	ER membrane protein complex subunit 1	2	71.0	0.031930	3.79
Q8VDN2	Sodium/potassium-transporting ATPase subunit alpha-1	6	442.1	0.043888	3.73
P10107	Annexin A1	19	1011.6	0.002737	3.70
O55026	Ectonucleoside triphosphate diphosphohydrolase 2	4	197.2	0.008935	3.68
P50427	Steryl-sulfatase	3	223.1	0.007529	3.64
P35564	Calnexin	7	399.6	0.012743	3.63
P29621	Serine protease inhibitor A3C	2	251.0	0.003331	3.62
Q91X72	Hemopexin	15	602.3	0.001611	3.61
Q9EQ06	Estradiol 17-beta-dehydrogenase 11	2	53.8	0.031562	3.60
Q60634	Flotillin-2	7	198.8	0.018138	3.59

Table S2. *Cont.*

Accession No.	Protein Name	Peptide Count	Confidence Score	Anova (*p*)	Fold Change
Q62188	Dihydropyrimidinase-related protein 3	2	79.0	0.000619	3.51
Q80WJ7	Protein LYRIC	3	108.8	0.010382	3.46
Q8R127	Saccharopine dehydrogenase-like oxidoreductase	2	53.7	0.018803	3.44
Q3UMR5	Calcium uniporter protein, mitochondrial	5	452.2	0.007146	3.43
P07759	Serine protease inhibitor A3K	9	659.2	0.007933	3.43
Q8BJS4	SUN domain-containing protein 2	2	130.8	0.003953	3.42
Q9Z1W8	Potassium-transporting ATPase alpha chain 2	2	167.9	0.032004	3.41
Q9D1G1	Ras-related protein Rab-1B	2	53.1	0.025936	3.35
P31428	Dipeptidase 1	3	128.4	0.015513	3.34
Q62465	Synaptic vesicle membrane protein VAT-1 homolog	4	133.9	0.001136	3.29
Q61543	Golgi apparatus protein 1	5	229.1	0.013191	3.25
Q9D666	SUN domain-containing protein 1	2	45.0	0.001556	3.25
P29788	Vitronectin	5	263.7	0.000396	3.23
P20918	Plasminogen	2	110.0	0.018958	3.20
Q9D783	Kelch-like protein 40	3	152.4	0.004784	3.19
P99024	Tubulin beta-5 chain	5	503.4	0.000555	3.18
Q61830	Macrophage mannose receptor 1	27	1385.3	0.000607	3.11
P70452	Syntaxin-4	3	109.9	0.004505	3.09
Q61838	Alpha-2-macroglobulin	29	1607.5	0.000155	3.09
Q9DBH5	Vesicular integral-membrane protein VIP36	5	172.6	0.011157	3.07
O35969	Guanidinoacetate N-methyltransferase	2	84.8	0.001370	3.07
Q64310	Surfeit locus protein 4	4	298.3	0.038601	3.06
Q8BK84	Dual specificity phosphatase DUPD1	3	257.0	0.001066	3.05
Q9WVA4	Transgelin-2	3	192.0	0.000434	3.04
O09161	Calsequestrin-2	3	173.4	0.016830	3.03
Q71LX4	Talin-2	2	48.9	0.003565	3.02
B2RSH2	Guanine nucleotide-binding protein G(i) subunit alpha-1	4	165.7	0.019610	2.97
Q61207	Sulfated glycoprotein 1	3	166.7	0.004729	2.96
Q6PIE5	Sodium/potassium-transporting ATPase subunit alpha-2	21	1375.1	0.017031	2.93
Q9QUI0	Transforming protein RhoA	2	102.6	0.011610	2.92
P22599	Alpha-1-antitrypsin 1-2	4	271.0	0.001990	2.92
Q61147	Ceruloplasmin	3	177.5	0.002923	2.91
P61027	Ras-related protein Rab-10	4	184.5	0.019421	2.91
P28656	Nucleosome assembly protein 1-like 1	2	93.2	0.001014	2.89
P97449	Aminopeptidase N	14	965.1	0.003192	2.87
P97429	Annexin A4	14	1308.6	0.000114	2.87
Q60770	Syntaxin-binding protein 3	2	68.0	0.001604	2.86
Q8BW75	Amine oxidase [flavin-containing] B	3	164.0	0.008239	2.85
P50543	Protein S100-A11	2	156.9	0.001067	2.84
O08917	Flotillin-1	11	769.2	0.000308	2.83
P35293	Ras-related protein Rab-18	6	330.2	0.026323	2.82
Q6P5E4	UDP-glucose:glycoprotein glucosyltransferase 1	10	511.3	0.005885	2.80
Q8VDL4	ADP-dependent glucokinase	2	112.7	0.005399	2.79
P07758	Alpha-1-antitrypsin 1-1	14	1113.8	0.004016	2.79

Table S2. *Cont.*

Accession No.	Protein Name	Peptide Count	Confidence Score	Anova (*p*)	Fold Change
Q9WVK4	EH domain-containing protein 1	2	51.1	0.000614	2.72
P28063	Proteasome subunit beta type-8	2	97.6	0.001538	2.70
P62821	Ras-related protein Rab-1A	2	133.2	0.022868	2.69
Q6PHN9	Ras-related protein Rab-35	3	174.0	0.015318	2.67
P08003	Protein disulfide-isomerase A4	3	181.1	0.009967	2.64
O08529	Calpain-2 catalytic subunit	4	124.7	0.003628	2.64
P16675	Lysosomal protective protein	3	98.2	0.007661	2.62
Q9D0F3	Protein ERGIC-53	3	195.3	0.000296	2.62
Q9EQK5	Major vault protein	27	1951.9	0.000392	2.61
P26041	Moesin	9	501.0	0.000491	2.60
Q07079	Insulin-like growth factor-binding protein 5	4	183.7	8.77E-05	2.60
P14733	Lamin-B1	2	43.9	0.013669	2.60
P62962	Profilin-1	7	417.6	0.000737	2.58
P97765	WW domain-binding protein 2	2	104.4	0.002495	2.58
Q8BH64	EH domain-containing protein 2	3	157.9	0.003089	2.56
P19324	Serpin H1	10	409.5	0.003513	2.56
Q8K273	Membrane magnesium transporter 1	2	126.1	0.020175	2.56
P01872	Ig mu chain C region	5	367.3	0.009373	2.55
Q922R8	Protein disulfide-isomerase A6	7	311.1	0.001110	2.54
Q07076	Annexin A7	11	602.3	0.000181	2.54
P14094	Sodium/potassium-transporting ATPase subunit beta-1	11	643.3	0.003179	2.54
P48036	Annexin A5	13	1064.6	0.002893	2.52
P24369	Peptidyl-prolyl cis-trans isomerase B	4	266.8	0.000249	2.51
Q61334	B-cell receptor-associated protein 29	3	207.2	0.006147	2.50
P42208	Septin-2	2	53.8	0.009304	2.49
P20152	Vimentin	19	1180.9	0.002422	2.47
P32261	Antithrombin-III	2	120.1	0.025224	2.46
Q61335	B-cell receptor-associated protein 31	2	288.5	0.003516	2.46
P60766	Cell division control protein 42 homolog	5	252.5	0.010639	2.45
P61979	Heterogeneous nuclear ribonucleoprotein K	5	325.9	0.001316	2.44
P16546	Spectrin alpha chain, non-erythrocytic 1	4	242.2	0.001063	2.44
P51150	Ras-related protein Rab-7a	7	228.8	0.016446	2.43
P29391	Ferritin light chain 1	14	1094.6	0.001140	2.42
P24549	Retinal dehydrogenase 1	6	182.4	0.006477	2.42
Q9Z1E4	Glycogen [starch] synthase, muscle	3	208.5	0.030318	2.41
P62746	Rho-related GTP-binding protein RhoB	2	88.5	0.021120	2.40
Q61475	Complement decay-accelerating factor, GPI-anchored	3	126.4	0.009179	2.39
P21614	Vitamin D-binding protein	4	111.0	0.017952	2.39
P70302	Stromal interaction molecule 1	13	989.1	0.000960	2.37
P16045	Galectin-1	7	649.3	0.000405	2.37
P09055	Integrin beta-1	9	617.5	0.001226	2.36
O70252	Heme oxygenase 2	3	101.1	0.004367	2.36
P17047	Lysosome-associated membrane glycoprotein 2	2	46.1	7.84E-05	2.36
Q6ZWV3	60S ribosomal protein L10	6	261.0	0.013502	2.36

Table S2. *Cont.*

Accession No.	Protein Name	Peptide Count	Confidence Score	Anova (*p*)	Fold Change
Q64727	Vinculin	13	851.5	0.000227	2.36
Q9CVB6	Actin-related protein 2/3 complex subunit 2	4	200.1	0.004120	2.33
A2AMM0	Muscle-related coiled-coil protein	5	293.7	0.004820	2.33
Q8VCM8	Nicalin	4	356.7	0.013996	2.32
P62835	Ras-related protein Rap-1A	8	470.6	0.017144	2.31
P01887	Beta-2-microglobulin	3	164.1	0.000290	2.30
Q6GQT9	Nodal modulator 1	10	566.3	0.008738	2.30
Q9DBG6	Dolichyl-diphosphooligosaccharide-protein glycosyltransferase SU-2	7	471.1	0.012763	2.29
Q91ZX7	Prolow-density lipoprotein receptor-related protein 1	3	169.6	0.006003	2.26
P18760	Cofilin-1	6	306.6	0.010858	2.22
Q9DB73	NADH-cytochrome b5 reductase 1	7	427.2	0.003680	2.21
Q91VI7	Ribonuclease inhibitor	6	382.7	6.54E-05	2.21
Q99P72	Reticulon-4	5	421.3	0.008373	2.21
P13020	Gelsolin	6	452.3	0.012795	2.19
Q99KV1	DnaJ homolog subfamily B member 11	3	139.2	0.008446	2.19
P07356	Annexin A2	19	1500.3	0.002916	2.17
Q6ZQI3	Malectin	5	156.3	0.026901	2.17
P63001	Ras-related C3 botulinum toxin substrate 1	3	83.9	0.004156	2.16
P27773	Protein disulfide-isomerase A3	19	1063.9	0.005435	2.16
Q60605	Myosin light polypeptide 6	2	107.2	0.015891	2.16
Q61316	Heat shock 70 kDa protein 4	3	157.5	0.001483	2.15
Q01339	Beta-2-glycoprotein 1	7	320.8	0.030850	2.14
Q8BFZ9	Erlin-2	2	75.6	0.043537	2.13
P51885	Lumican	7	426.4	0.019648	2.13
P07309	Transthyretin	5	504.8	0.020417	2.12
P09103	Protein disulfide-isomerase	19	1223.2	0.018494	2.12
Q9JKR6	Hypoxia up-regulated protein 1	10	568.5	0.009349	2.12
Q8VEH8	Endoplasmic reticulum lectin 1	2	85.8	0.010297	2.11
Q8C7E7	Starch-binding domain-containing protein 1	3	61.1	0.003248	2.10
P55772	Ectonucleoside triphosphate diphosphohydrolase 1	2	7.6	0.045918	2.10
O88384	Vesicle transport through interaction with t-SNAREs homolog 1B	2	64.0	0.000786	2.10
P26645	Myristoylated alanine-rich C-kinase substrate	2	108.5	0.001837	2.09
Q9DBC7	cAMP-dependent protein kinase type I-alpha regulatory subunit	5	187.3	0.007118	2.08
Q9JII6	Alcohol dehydrogenase [NADP(+)]	2	104.9	0.003829	2.07
Q01730	Ras suppressor protein 1	2	157.2	0.016782	2.06
O08677	Kininogen-1	5	101.1	0.004696	2.05
Q91YQ5	Dolichyl-diphosphooligosaccharide-protein glycosyltransferase SU-1	10	510.1	0.024405	2.04
P50516	V-type proton ATPase catalytic subunit A	3	191.7	0.000247	2.04

Table S2. *Cont.*

Accession No.	Protein Name	Peptide Count	Confidence Score	Anova (*p*)	Fold Change
Q04447	Creatine kinase B-type	3	147.0	0.015351	2.03
O54734	Dolichyl-diphosphooligosaccharide-protein glycosyltransferase 48 kDa	3	116.3	0.017382	2.03
Q91V41	Ras-related protein Rab-14	3	164.7	0.027554	2.03
P63158	High mobility group protein B1	4	315.3	0.005203	2.01
P62814	V-type proton ATPase subunit B, brain isoform	3	185.2	0.000798	2.01
Q05816	Fatty acid-binding protein, epidermal	2	44.2	0.010133	2.00

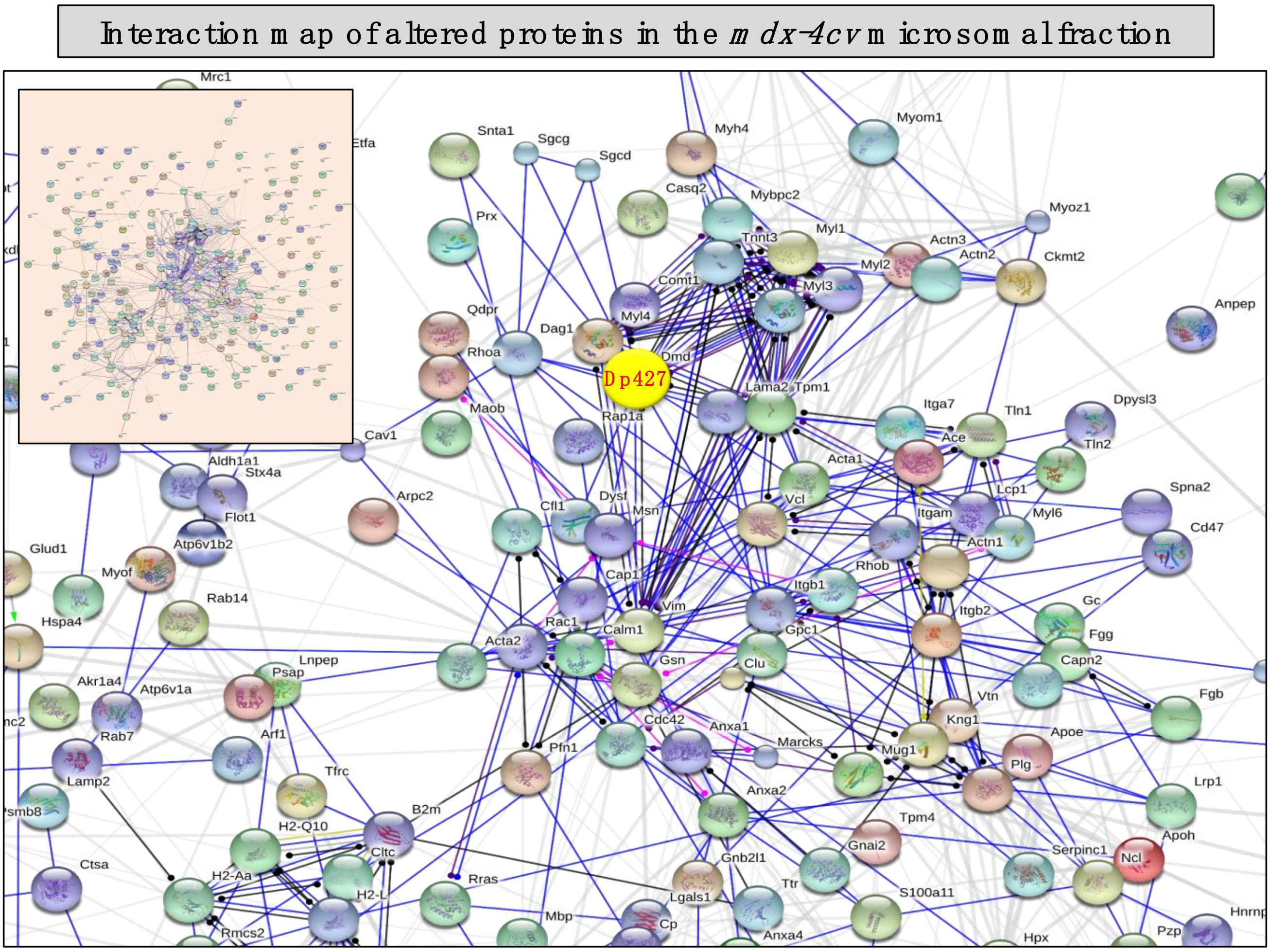

Figure S1. Interaction map of altered microsomal proteins from *mdx-4cv* hind limb muscle. The bioinformatics STRING database was applied to generate a protein interaction map with known and predicted protein associations that include direct physical and indirect functional protein linkages. The analysis of mass spectrometrically identified proteins with a changed abundance in *mdx-4cv* muscle was based on the proteomic data presented in Tables 1, 2, S1 and S2. The insert shows the entire interaction map and the main panel focuses on the central position of the dystrophin protein Dp427 (marked in yellow).

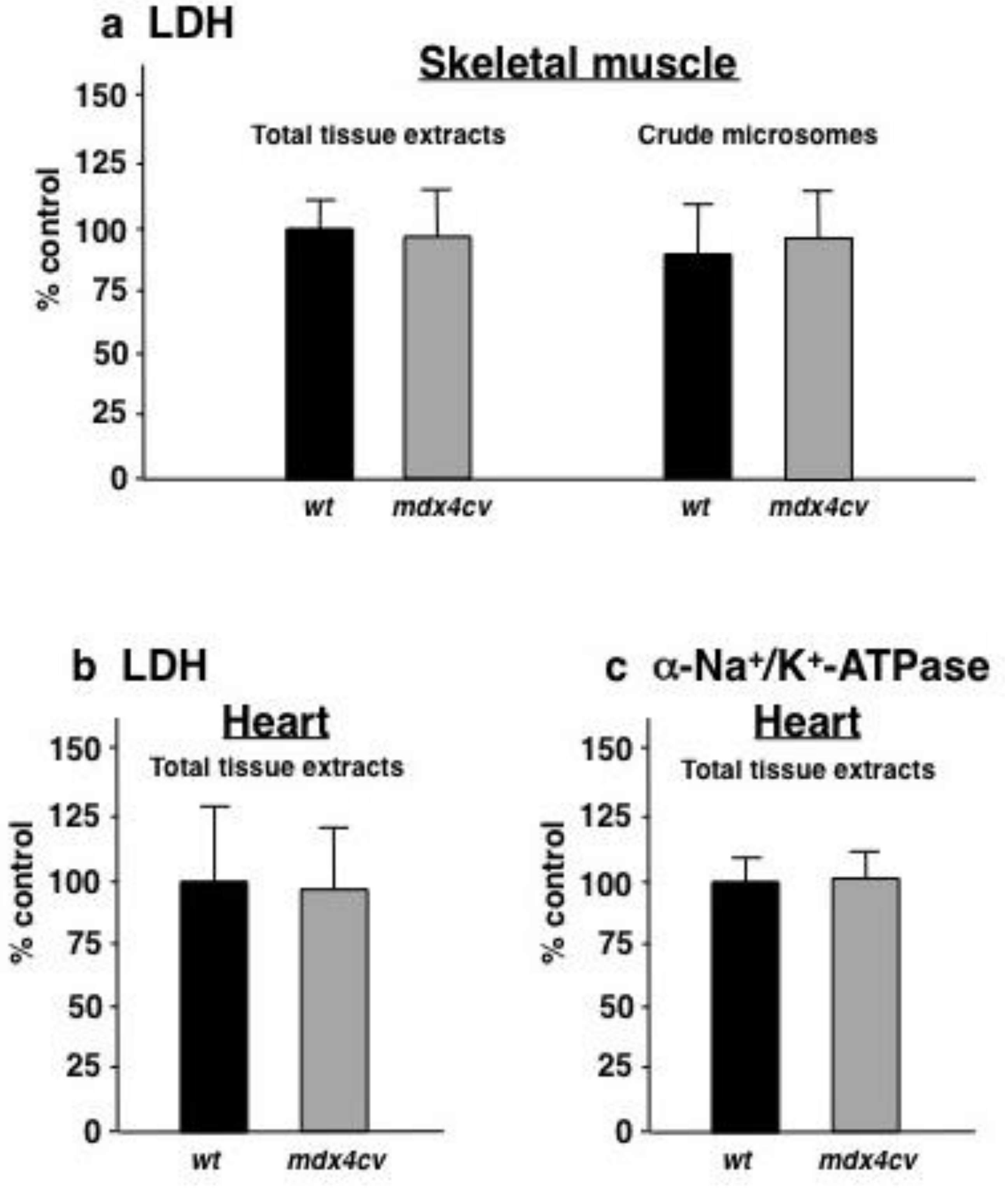

Figure S2. Graphical presentation of the control immunoblots shown in Figure 6, which displays primarily the immunoblot analysis of the Na^+/K^+-ATPase from *mdx-4cv* skeletal muscle. Shown are graphs of the immunoblotting results using antibodies to lactate dehydrogenase (LDH) (**a**), (**b**), as a loading control of both total tissue extracts and the crude microsomal preparations, and the cardiac Na^+/K^+-ATPase (**c**) in normal versus *mdx-4cv* preparations.

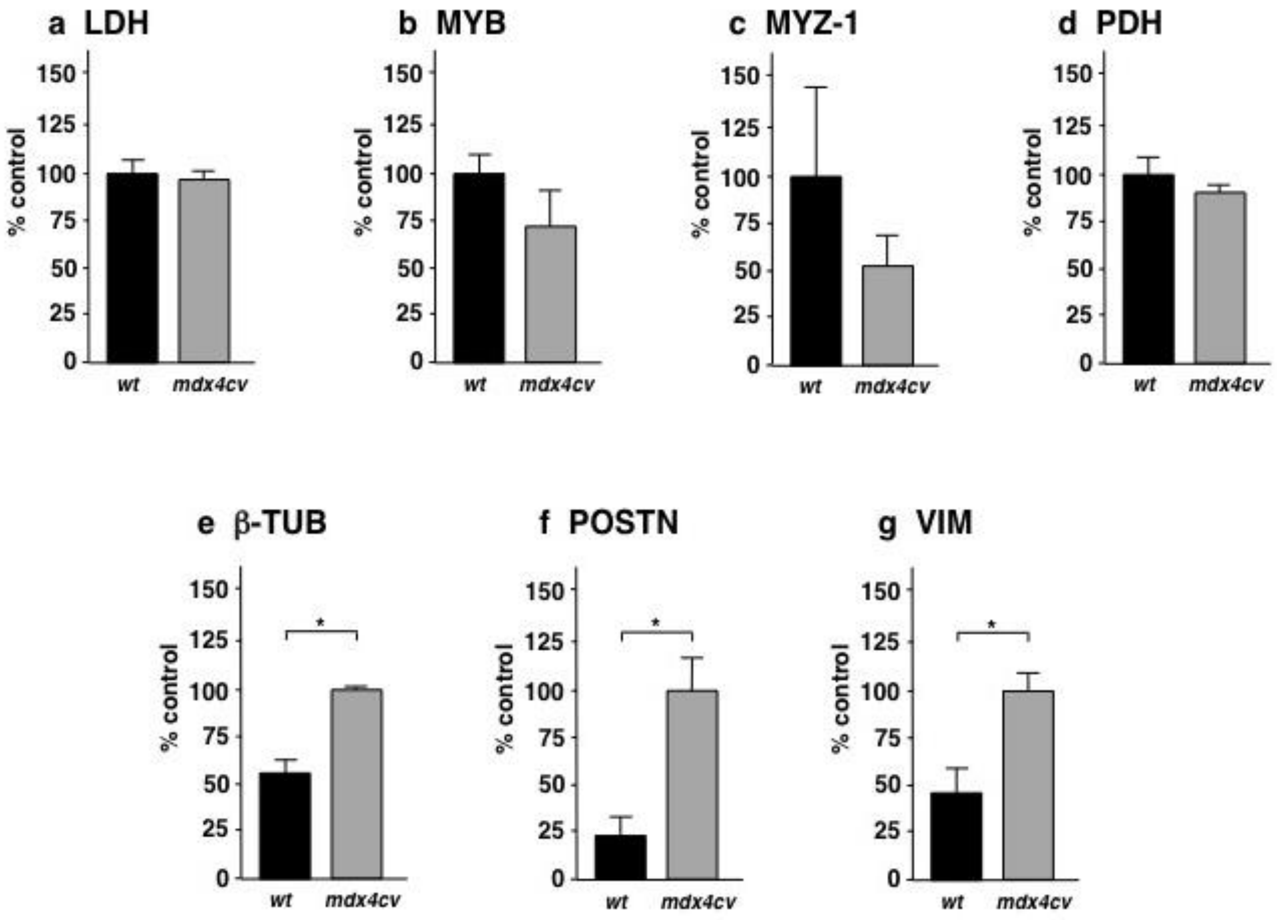

Figure S3. Graphical presentation of the immunoblotting survey of proteins with a changed abundance in *mdx-4cv* skeletal muscle tissue, presented in Figure 8. Shown are graphs of the immunoblotting results using antibodies to lactate dehydrogenase (LDH) (**a**), myoglobin (MYB) (**b**), myozenin (MYZ-1) (**c**), pyruvate dehydrogenase (PDH) (**d**), β-tubulin (β-TUB) (**e**), periostin (POSTN) (**f**) and vimentin (VIM) (**g**). Lanes 1 and 2 represent crude microsomes from wild type (*wt*) muscle and dystrophic *mdx-4cv* muscle, respectively. Student's *t*-test, unpaired; $n = 4$; * $p < 0.05$.
